# Complete genome sequence of the thermophilic, hydrogen-oxidizing *Bacillus tusciae* type strain (T2^T^) and reclassification in the new genus, *Kyrpidia* gen. nov. as *Kyrpidia tusciae* comb. nov. and emendation of the family *Alicyclobacillaceae* da Costa and Rainey, 2010.

**DOI:** 10.4056/sigs.2144922

**Published:** 2011-09-23

**Authors:** Hans-Peter Klenk, Alla Lapidus, Olga Chertkov, Alex Copeland, Tijana Glavina Del Rio, Matt Nolan, Susan Lucas, Feng Chen, Hope Tice, Jan-Fang Cheng, Cliff Han, David Bruce, Lynne Goodwin, Sam Pitluck, Amrita Pati, Natalia Ivanova, Konstantinos Mavromatis, Chris Daum, Amy Chen, Krishna Palaniappan, Yun-juan Chang, Miriam Land, Loren Hauser, Cynthia D. Jeffries, John C. Detter, Manfred Rohde, Birte Abt, Rüdiger Pukall, Markus Göker, James Bristow, Victor Markowitz, Philip Hugenholtz, Jonathan A. Eisen

**Affiliations:** 1 DSMZ – German Collection of Microorganisms and Cell Cultures GmbH, Braunschweig, Germany; 2 DOE Joint Genome Institute, Walnut Creek, California, USA; 3 Los Alamos National Laboratory, Bioscience Division, Los Alamos, New Mexico, USA; 4 Biological Data Management and Technology Center, Lawrence Berkeley National Laboratory, Berkeley, California, USA; 5 Oak Ridge National Laboratory, Oak Ridge, Tennessee, USA; 6 HZI – Helmholtz Centre for Infection Research, Braunschweig, Germany; 7 Australian Centre for Ecogenomics, School of Chemistry and Molecular Biosciences, The University of Queensland, Brisbane, Australia; 8 University of California Davis Genome Center, Davis, California, USA

**Keywords:** hydrogen-oxidizing, aerobe, facultative chemolithoautotroph, thermoacidophile, free-living, solfatara, spore-forming, *Bacillaceae*, GEBA

## Abstract

*Bacillus tusciae* Bonjour & Aragno 1994 is a hydrogen-oxidizing, thermoacidophilic spore former that lives as a facultative chemolithoautotroph in solfataras. Although 16S rRNA gene sequencing was well established at the time of the initial description of the organism, 16S sequence data were not available and the strain was placed into the genus *Bacillus* based on limited chemotaxonomic information. Despite the now obvious misplacement of strain T2 as a member of the genus *Bacillus* in 16S rRNA-based phylogenetic trees, the misclassification remained uncorrected for many years, which was likely due to the extremely difficult, analysis-hampering cultivation conditions and poor growth rate of the strain. Here we provide a taxonomic re-evaluation of strain T2T (= DSM 2912 = NBRC 15312) and propose its reclassification as the type strain of a new species, *Kyrpidia tusciae*, and the type species of the new genus *Kyrpidia*, which is a sister-group of *Alicyclobacillus*. The family *Alicyclobacillaceae* da Costa and Rainey, 2010 is emended. The 3,384,766 bp genome with its 3,323 protein-coding and 78 RNA genes is part of the *** G****enomic* *** E****ncyclopedia of* *** B****acteria and* *** A****rchaea * project.

## Introduction

Strain T2^T^ (= DSM 2912 = NBRC 15312) is the type strain of the *Bacillus tusciae* [1]. Both strain T2^T^ and strain T201 were isolated from ponds in the solfatara of San Federigo, a geothermal area near Lago, Tuscany, Italy [1,Table 1], as indicated in the Latin species epithet *tusciae*, meaning from Tuscia [1]. In the original study of Bonjour and Aragno [1], DNA:DNA reassociation studies of strains T2^T^, *Bacillus schlegelii* (also autotrophic) and the thermoacidophilic *Bacillus acidocaldarius* (later on reclassified as *Alicyclobacillus acidocaldarius*) from another hot spring were carried out. Because of the low levels of hybridization observed, the authors proposed that strains T2^T^ and T201 formed a distinct taxonomic unit [1]. However, only limited chemotaxonomic data was available at the time and 16S rRNA sequence data was not available, which led the authors to place the species into the genus *Bacillus* [1]. Rather difficult cultivation conditions and the poor growth rate of strain T2^T^ likely delayed an earlier re-classification although Rainey *et al*. previously noted the link to the genus *Alicyclobacillus* [19]. This was recently corroborated by the observation that strain T2^T^ as well as *A. acidocaldarius* lack the *sspE* gene for acid-soluble spore proteins frequently found in members of the *Bacillales* [20]. Here we present a summary classification and a set of features for *B. tusciae* strain T2^T^, a description of the complete genome sequencing and annotation and a proposal to reclassify *B. tusciae* as a member of the new genus *Kyrpidia* as *Kyrpidia tuscae* comb. nov.

**Table 1:** Classification and general features of K*yrpidia tusciae* strain T2^T^ according to the MIGS recommendations [2] and the NamesforLife database [3].

**MIGS ID**	**Property**	**Term**	**Evidence code**
	Current classification	Domain *Bacteria*	TAS [4]
		Phylum *Firmicutes*	TAS [5-7]
		Class *Bacilli*	TAS [8,9]
		Order *Bacillales*	TAS [10,11]
		Family *Bacillaceae*	TAS [10,12]
		Genus *Bacillus*	TAS [10,13,14]
		Species *Bacillus tusciae*	TAS [1,15]
		Type strain T2	TAS [1]
			
	Revised classification	Family *Alicyclobacillaceae*	TAS [9,16]
		Genus *Kyrpidia*	NAS
		Species *Kyrpidia tusciae*	NAS
	Gram stain	positive	TAS [1]
	Cell shape	straight rods	TAS [1]
	Motility	not reported, but lateral flagella visible	TAS [1]
	Sporulation	sporulating	TAS [1]
	Temperature range	thermophile, grows > 47°, < 65°C	TAS [1]
	Optimum temperature	55°C	TAS [1]
	Salinity	not reported	
MIGS-22	Oxygen requirement	aerobic	TAS [1]
	Carbon source	short chain fatty acids, amino acids and alcohols	TAS [1]
	Energy metabolism	facultatively chemolithoautotroph	TAS [1]
MIGS-6	Habitat	hot, acidic solfatara fields	TAS [1]
MIGS-15	Biotic relationship	free living	TAS [1]
MIGS-14	Pathogenicity	none	NAS
	Biosafety level	1	TAS [17]
	Isolation	ponds in solfatara	TAS [1]
MIGS-4	Geographic location	San Frederigo, near Lago, Tuscany (Italy)	TAS [1]
MIGS-5	Sample collection time	about or before 1984	TAS [1]
MIGS-4.1 MIGS-4.2	Latitude Longitude	43.33 10.50	NAS
MIGS-4.3	Depth	not reported	
MIGS-4.4	Altitude	about 155 m	NAS

Evidence codes - TAS: Traceable Author Statement (i.e., a direct report exists in the literature); NAS: Non-traceable Author Statement (i.e., not directly observed for the living, isolated sample, but based on a generally accepted property for the species, or anecdotal evidence). These evidence codes are from of the Gene Ontology project [18].

## Classification and features

A representative genomic 16S rRNA sequence of *B. tusciae* T2^T^ was compared using NCBI BLAST [21] under default settings (e.g., considering only the high-scoring segment pairs (HSPs) from the best 250 hits) with the most recent release of the Greengenes database [22] and the relative frequencies of taxa and keywords (reduced to their stems [23]) were determined, weighted by BLAST scores. The most frequently occurring genera were *Alicyclobacillus* (67.8%), *Bacillus* (18.2%), *Thermoactinomyces* (6.0%), *Paenibacillus* (5.6%) and *Exiguobacterium* (1.6%) (99 hits in total). Regarding the seven hits to sequences from members of the species, the average identity within HSPs was 99.6%, whereas the average coverage by HSPs was 99.5%. Among all other species, the one yielding the highest score was *A. acidiphilus* NR_028637, which corresponds to an identity of 92.4% and an HSP coverage of 57.7%. (Note that the Greengenes database uses the INSDC (= EMBL/NCBI/DDBJ) annotation, which is not an authoritative source for nomenclature or classification.) The highest-scoring environmental sequence was EU638396 ('Ecological Role Firmicutes Identified Thermophilic Microbial Fuel Cells thermophilic microbial fuel cell acetate-fed experiment clone SHBZ1905')), which showed an identity of 99.4% and an HSP coverage of 90.0%. The most frequently occurring keywords within the labels of all environmental samples that were hits were 'microbi' (13.3%), 'thermophil' (12.5%), 'cell, fuel' (12.4%), 'ecolog, firmicut, identifi, role' (6.2%) and 'experi' (6.1%) (151 hits in total). The most frequently occurring keywords within the labels of those environmental samples which yielded hits of a higher score than the highest scoring species were 'microbi' (13.9%), 'cell, fuel, thermophil' (13.1%), 'ecolog, firmicut, identifi, role' (6.5%), 'experi' (6.4%) and 'acetate-f' (4.7%) (124 hits in total). These keywords corroborate the features of the environment from which strain T2^T^ was isolated.

**Figure 1:**
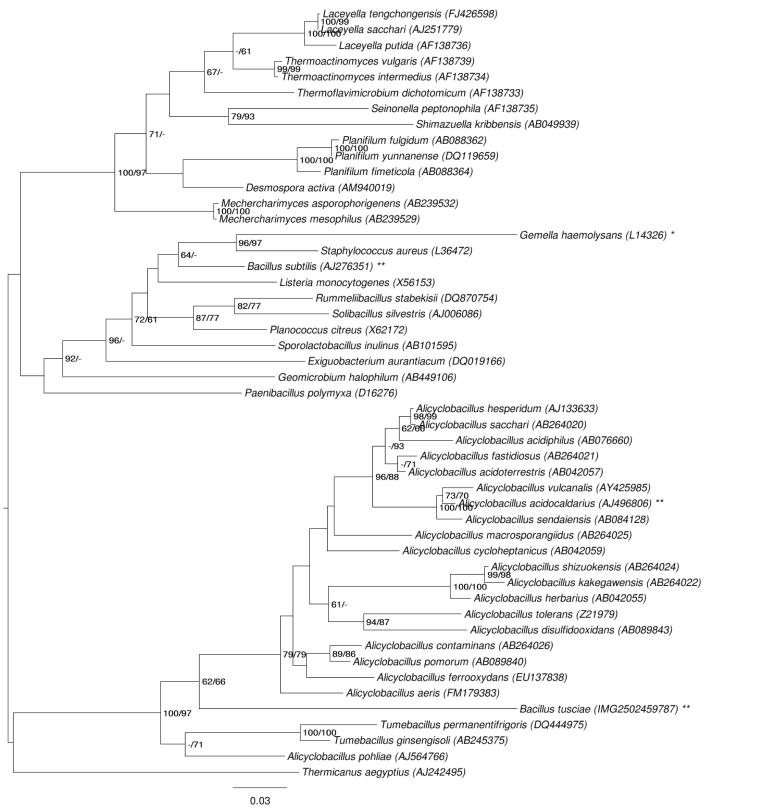
Phylogenetic tree highlighting the position of *B. tusciae* relative to the type strains within the families *Alicyclobacillaceae* and *Thermoactinomycetaceae*, which gave the best hits when conducting a BLAST search [22] against the sequences contained in the last release from the All-Species-Living-Tree Project [24], and the type strains of all other families within the order *Bacillales*. The tree was inferred from 1,403 aligned characters [25,26] of the 16S rRNA gene sequence under the maximum likelihood (ML) criterion [27]. Rooting was done initially using the midpoint method [28] and then checked for its agreement with the current classification (Table 1). The branches are scaled in terms of the expected number of substitutions per site. Numbers adjacent to the branches are support values from 450 ML bootstrap replicates [29] (left) and from 1,000 maximum parsimony (MP) bootstrap replicates [30] (right) if larger than 60%. Lineages with type strain genome sequencing projects registered in GOLD [31] are labeled with one asterisk, those also listed as 'Complete and Published' with two asterisks [32,33].

Figure 1 shows the phylogenetic neighborhood of *B. tusciae* in a 16S rRNA tree. The sequences of the five 16S rRNA gene copies in the genome differ from each other by up to eight nucleotides, and differ by up to six nucleotides from the previously published 16S rRNA sequence AB042062.

**Figure 2:**
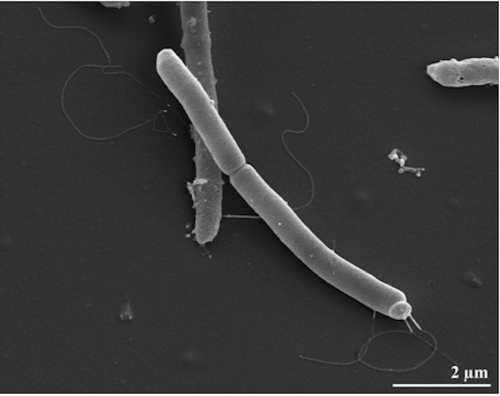
Scanning electron micrograph of *B. tusciae* strain T2^T^

*B. tusciae* cells are straight rods measuring 0.8 x 4-5 μm length (Figure 2) with single laterally inserted flagella [1]. In young cultures, cells stain Gram-positive and exhibit oval, subterminal spores that swell the sporangium [1]. Cultures grow at 55°C at a pH ranging from 4.2 to 4.8 [1]. Although cells grow best under autotrophic conditions *via* hydrogen oxidation, they can also grow heterotrophic on alcohols, amino acids and short chain fatty acids, but not with sugars [1]. Substrate usage was described in detail by Bonjour and Aragno [1]. Cells contain a soluble malate-dehydrogenase activity, which cannot reduce pyridine (NAD^+^/NAD(P)^+^) [1], nor NADH oxidation was observed [1]. Hydrogenase activity was reported as being inducible [1]. An operational Calvin cycle was reported based on the presence of ribulose-1,5-bisphosphate carboxylase activity in autotrophically-grown cells [1]. Autotrophically grown cells show inclusions of poly-β-hydroxybutyric acid [1].

### Chemotaxonomy

The structure of the cell wall of strain T2^T^ was reported as typically Gram-positive, without a protein coat [1]. ω-alicyclic fatty acids are lacking and menaquinone MK-7 was identified as the main quinone (personal communication Brian J. Tindall).

## Genome sequencing and annotation

### Genome project history

This organism was selected for sequencing on the basis of its phylogenetic position [34], and is part of the *** G****enomic* *** E****ncyclopedia of* *** B****acteria and* *** A****rchaea * project [35]. The genome project is deposited in the Genomes OnLine Database [31] and the complete genome sequence in GenBank. Sequencing, finishing and annotation were performed by the DOE Joint Genome Institute (JGI). A summary of the project information is shown in Table 2.

**Table 2:** Genome sequencing project information

**MIGS ID**	**Property**	**Term**
MIGS-31	Finishing quality	Finished
MIGS-28	Libraries used	Two genomic libraries: Sanger 8 kb pMCL200 and 454 standard library
MIGS-29	Sequencing platforms	ABI3730, 454 GS FLX
MIGS-31.2	Sequencing coverage	5.5 × Sanger; 25.2 × pyrosequence
MIGS-30	Assemblers	Newbler version 2.0.0-PreRelease-07/15/2008, phrap
MIGS-32	Gene calling method	Prodigal 1.4, GenePRIMP
	Genbank ID	CP002017
	Genbank Date of Release	April 5, 2010
	GOLD ID	Gc01268
	NCBI project ID	31345
	Database: IMG-GEBA	646564511
MIGS-13	Source material identifier	DSM 2912
	Project relevance	Tree of Life, GEBA

### Growth conditions and DNA isolation

*B. tusciae* strain T2^T^, DSM 2912, was grown in DSMZ medium 369 (*Bacillus tusciae* medium) [36] at 50°C. DNA was isolated from 1-1.5 g of cell paste using Qiagen Genomic 500 DNA Kit (Qiagen, Hilden, Germany) with lysis modification st/LALMP according to Wu *et al*. [35]. DNA is available through the DNA Bank Network [37].

### Genome sequencing and assembly

The genome was sequenced using a combination of Sanger and 454 sequencing platforms. All general aspects of library construction and sequencing can be found at the JGI website [38]. Pyrosequencing reads were assembled using the Newbler assembler (Roche). Large Newbler contigs were broken into 3,650 overlapping fragments of 1,000 bp and entered into assembly as pseudo-reads. The sequences were assigned quality scores based on Newbler consensus q-scores with modifications to account for overlap redundancy and adjust inflated q-scores. A hybrid 454/Sanger assembly was made using the phrap assembler [39]. Possible mis-assemblies were corrected with Dupfinisher and gaps between contigs were closed by editing in Consed, by custom primer walks from sub-clones or PCR products [40]. A total of 549 Sanger finishing reads were needed to close gaps, to resolve repetitive regions, and to raise the quality of the finished sequence. The error rate of the completed genome sequence is less than 1 in 100,000. Together, the combination of the Sanger and 454 sequencing platforms provided 30.7 × coverage of the genome. The final assembly contains 18,870 Sanger reads and 413,112 pyrosequencing reads.

### Genome annotation

Genes were identified using Prodigal [41] as part of the Oak Ridge National Laboratory genome annotation pipeline, followed by a round of manual curation using the JGI GenePRIMP pipeline [42]. The predicted CDSs were translated and used to search the National Center for Biotechnology Information (NCBI) non-redundant database, UniProt, TIGR-Fam, Pfam, PRIAM, KEGG, COG, and InterPro databases. Additional gene prediction analysis and functional annotation was performed within the Integrated Microbial Genomes - Expert Review platform [43].

## Genome properties

**Figure 3:**
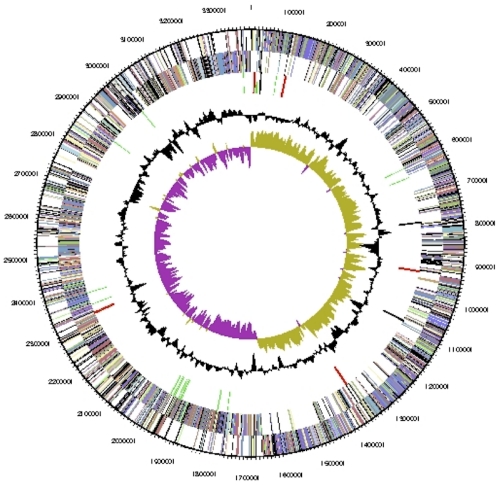
Graphical circular map of the chromosome. From outside to the center: Genes on forward strand (color by COG categories), Genes on reverse strand (color by COG categories), RNA genes (tRNAs green, rRNAs red, other RNAs black), GC content, GC skew.

The genome consists of a 3,384,766 bp long circular chromosome with a G+C content of 59.1% (Table 3 and Figure 3). Of the 3,401 genes predicted, 3,323 were protein-coding genes, and 78 RNAs; 173 pseudogenes were also identified. The majority of the protein-coding genes (70.7%) were assigned a putative function while the remaining ones were annotated as hypothetical proteins. The distribution of genes into COGs functional categories is presented in Table 4.

**Table 3:** Genome Statistics

**Attribute**	**Value**	**% of Total**
Genome size (bp)	3,384,766	100.00%
DNA coding region (bp)	2,896,588	85.58%
DNA G+C content (bp)	2,000,875	59.11%
Number of replicons	1	
Extrachromosomal elements	0	
Total genes	3,401	100.00%
RNA genes	78	2.29%
rRNA operons	5	
Protein-coding genes	3,323	97.71%
Pseudo genes	173	5.09%
Genes with function prediction	2,404	70.69%
Genes in paralog clusters	718	21.11%
Genes assigned to COGs	2,456	74.21%
Genes assigned Pfam domains	2,657	78.12%
Genes with signal peptides	530	15.58%
Genes with transmembrane helices	728	21.41%
CRISPR repeats	4	

**Table 4:** Number of genes associated with the general COG functional categories

**Code**	**value**	**%age**	**Description**
J	153	5.6	Translation, ribosomal structure and biogenesis
A	0	0.0	RNA processing and modification
K	198	7.2	Transcription
L	192	7.0	Replication, recombination and repair
B	1	0.0	Chromatin structure and dynamics
D	36	1.3	Cell cycle control, cell division, chromosome partitioning
Y	0	0.0	Nuclear structure
V	44	1.6	Defense mechanisms
T	121	4.4	Signal transduction mechanisms
M	116	4.2	Cell wall/membrane/envelope biogenesis
N	62	2.3	Cell motility
Z	1	0.0	Cytoskeleton
W	0	0.0	Extracellular structures
U	41	1.5	Intracellular trafficking, secretion and vesicular transport
O	95	3.5	Posttranslational modification, protein turnover, chaperones
C	217	7.9	Energy production and conversion
G	111	4.1	Carbohydrate transport and metabolism
E	255	9.3	Amino acid transport and metabolism
F	68	2.5	Nucleotide transport and metabolism
H	133	4.9	Coenzyme transport and metabolism
I	145	5.3	Lipid transport and metabolism
P	133	4.9	Inorganic ion transport and metabolism
Q	97	3.5	Secondary metabolites biosynthesis, transport and catabolism
R	298	10.9	General function prediction only
S	224	8.2	Function unknown
-	945	27.8	Not in COGs

## Insights into the genome

At the time when the species name *B. tusciae* was validly published [9], a 16S rRNA gene sequence of the strain was not generated, but DNA-DNA hybridizations (DDHs) were performed with two reference strains, *B. schlegelii* [44] and *B. acidocaldarius* [45], to show that strain T2^T^ represents a novel species within the genus *Bacillus* [1]. While *B. schlegelii* was named by the same senior author as *B. tusciae* and most probably is as misplaced in the genus *Bacillus* as *B. tusciae* (see below), *B. acidocaldarius* was subsequently reclassified as *Alicyclobacillus acidocaldarius* [46]. In retrospect, considering 16S rRNA gene sequence identities of 88.8% (for *A*. *acidocaldarius*) and 85.7% (for *B. schlegelii*) it is not surprising that Bonjour and Aragon observed only 17% and 0% DDH similarity, respectively, for their novel isolate [1]. While the members of the genus *Alicyclobacillus* do in fact represent the closest relatives of strain T2^T^ (Figure 1) with 87.5% to 89.0% 16S rRNA sequence identity in EzTaxon [47], the appropriate taxonomic placement of *B. schlegelii* seems to be an unresolved question, with no greater than 89.3% rRNA sequence identity found with other type strains, none of which are members of the genus *Bacillus* [47]. Figure 1 shows *B. tusciae* as a sister group of one of the two clades that comprise *Alicyclobacillus*, with *A. pohliae* more closely related to *Tumebacillus* than to the other members of the genus and likely being misclassified based on the current data. However, while the clade comprising *B. tusciae*, *Alicyclobacillus* and *Tumebacillus* has significant statistical support (100% under ML and 97% under MP), the claim that the clade comprises only *Tumebacillus* and *A. pohliae* is unsupported.

As two of the genera selected for Figure 1, *Alicyclobacillus* and *Bacillus*, appeared as polyphyletic in the tree, we conducted both unconstrained heuristic searches for the best tree under the ML [27] and MP criterion [30] as well as searches constrained for the monophyly of these two genera, respectively (for details of the data matrix see the figure caption). The best-known ML tree had a log likelihood of -13,289.73, whereas the best trees found under the constraint of *Alicyclobacillus* monophyly had a log likelihood of -13,297.23 and was not significantly worse in the Shimodaira-Hasegawa test as implemented in RAxML [27] (α = 0.05). However, when enforcing *Bacillus* monophyly, the resulting log likelihood was -13,412.24, significantly worse than the best tree (α = 0.01). The best-known MP trees had a score of 2,362, whereas the best trees found under the constraint of *Alicyclobacillus* monophyly had a score of 2,374 and were not significantly worse in the Kishino-Hasegawa test as implemented in PAUP* [30] (α = 0.05). When enforcing *Bacillus* (*Bacillus subtilis* and *B. tusciae*) monophyly, the resulting score was 2,439, significantly worse than the best tree (p < 0.0001). (See, e.g., chapter 21 in [48] for an in-depth description of such paired-site tests.). Accordingly, the current classification of *B. tusciae* in *Bacillus* is at odds with the 16S rRNA data and does not reflect the natural relationships based on that gene. In contrast, the placement of *A. pohliae* in *Alicyclobacillus* is not significantly disputed by the data.

**Figure 4:**
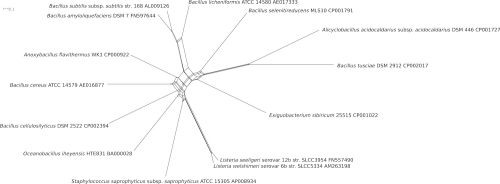
Unrooted phylogenetic network inferred with the Neighbor-Net algorithm as implemented in SplitsTree version 4.10 [52] from genome-to-genome distances calculated using GGDC [49-51]. The analysis includes all completely sequenced *Bacillales* type strain genomes as registered in GOLD at the time of publication [31]. Here, the logarithmic version of the distance calculated as the total genome length minus total number of identical base pairs within HSPs, divided by total genome length (see Table 5), was used as GGDC distance.

Table 5 shows the whole-genome distances between *B. tusciae*, *A. acidocaldarius* [32] and *B. subtilis* [33] as calculated using the genome-to-genome distance calculator [49-51]. The lower left triangle shows those distances derived by dividing the total sequence length not covered by HSPs through total genome length (left) and by dividing the total number of non-identical base pairs within HSPs by total HSP length (right); the upper right triangle shows the distance derived by dividing total genome length minus total number of identical base pairs within HSPs by total genome length. As expected, those distances relating HSP coverage and number of identical base pairs within HSPs to total genome length are higher between *B. tusciae* and *B. subtilis* than between *B. tusciae* and *A. acidocaldarius*. That the distances relating the number of identical base pairs to total HSP length behave differently indicates that the genomic similarities between *B. tusciae* and *B. subtilis* are more strongly restricted to more conserved sequences, a kind of saturation phenomenon [50]. Figure 4 shows an unrooted phylogenetic network inferred using the Neighbor-Net algorithm from whole-genome distances calculated with GGDC [49-51]. The grouping of *B. tusciae* and *A. acidocaldarius*, as well as the very tree-like appearance of this part of the network indicate that genomic data are also in conflict with the placement of *B. tusciae* within *Bacillus*.

**Table 5:** Genome-to-genome distances as calculated using GGDC [49-51].

	*B. tusciae*	*B. subtilis* subsp. *subtilis*	*A. acidocaldarius*
*B. tusciae* (CP002017 = NC_014098)	0.0000	0.9916	0.97030
*B. subtilis* subsp. *subtilis* (AL009126 = NC_000964)	0.9902/0.1452	0.0000	0.9908
*A. acidocaldarius* (CP001727-001730 = NC_013205, 07, 08)	0.9646/0.1629	0.9893/0.1366	0.0000

**Figure 5:**
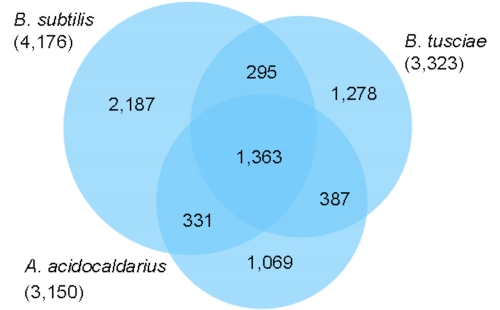
Venn diagram depicting the intersections of protein sets between the target genome and representative genomes of *Bacillus* and *Alicyclobacillus*.

The fraction of shared genes in the genomes of *B. tusciae* T2^T^, *A. acidocaldarius* [32] and *B. subtilis* [33] is shown in a Venn diagram (Figure 5). The numbers of pairwise shared genes were calculated with the phylogenetic profiler function of the IMG-ER platform [43]. The homologous genes within the genomes were detected with a maximum e-value of 10^-5^ and a minimum identity of 30%.

A total of 1,363 of the genes are shared by all three genomes, with about equal numbers of genes (295-387) shared between pairs of genomes to the exclusion of the third genome. Within the 1,278 unique genes of *B. tusciae* that have no detectable homologues in the genomes of *A. acidocaldarius* and *B. subtilis* (under the sequence similarity thresholds used for the comparison) are the genes encoding the key enzymes for the Calvin cycle: ribulose-1,5-bisphosphate carboxylase (Btus_2871 large subunit, Btus_2872 small subunit) and the phosphoribulokinase (Btus_2868). A closer look at the genome of *B. tusciae* revealed additional genes coding for enzymes of the Calvin cycle located in the immediate neighborhood of the ribulose-1,5-bisphosphate carboxylase genes: phosphoglyceratekinase (Btus_2865), fructose-bisphosphate aldolase (Btus_2867), phosphoribulokinase (Btus_2868), glyceraldehyde-3-phosphate dehydrogenase (Btus_2869), fructose-1,6-bisphosphatase (Btus_2870) ribulose-1,5-bisphosphate carboxylase (Btus_2871 large subunit, Btus_2872 small subunit) which are probably organized as an operon. In autotrophically-grown cells of *B. tusciae*, an active ribulose-1,5-bisphosphate carboxylase in an operating Calvin cycle was reported [1].

The phylogenetic position of strain T2^T^ as shown in Figures 1 and 4, corroborated by its primarily autotrophic energy metabolism [1] (Tables 1 and 6), and the lack of *sspE* genes for acid-soluble spore proteins [20] (Table 6), indicated a clear need to reclassify *B. tusciae*. The joint but distinct phylogenetic position (Figure 1) supports the establishment of a novel genus rather than the inclusion of *B. tusciae* in *Alicyclobacillus*. As shown above, 16S rRNA data do not indicate the monophyly of the remaining *Alicyclobacillus* spp., but are not in significant conflict with it. The branch leading to *B. tusciae* is rather long, indicating a considerable degree of independent evolution (relative to the other taxa included in the tree, which include the closest relatives of *B. tusciae* in the current LTP release, see above). Whether the lack of ω-alicyclic fatty acids in *A. pohliae* and perhaps other *Alicyclobacillus* spp. can be used in later studies for a revision of *Alicyclobacillus* remains to be seen.

**Table 6:** Typical features of reference taxa

	*Bacillus tusciae* T2^T^ DSM 2912 [1]	Genus *Alicyclobacillus* [53,54]	Genus *Bacillus* [54]
cell shape	straight rods, 4-5 μm long	rods, 1.5-6.3 μm long	rods, normally up to 5 µm long
Gram stain	positive	positive	positive
oxygen requirement	aerobic, facultative anaerobic, but not with nitrate	aerobic, facultative anaerobic	aerobic, facultative anaerobic, a few species are strictly anaerobic
endospores	subterminal, oval	terminal up to subterminal	ellipsoidal, central
*sspE* genes for acid-soluble spore proteins	missing	missing	frequent
growth temperature	>47°C to <67°C, opt. 55°C	4-70°C, opt. 35-65°C	10-60°C
pH optimum	4.2-4.8	< 4.5, (range 1.5-5)	5-10
phenotype	thermoacidophile	thermoacidophiles	some obligate or facultative thermophiles
habitat	ponds in solfatara of geothermal area	acidic geothermal sites (soil and water), fruit juices, ores	often saprophytes in decaying organic matter
flagellation	single lateral flagellum	motility reported for some species	motile or nonmotile; lateral
menaquinones	MK-7 (*personal communication Brian J. Tindall*)	MK-7	MK-7
major membranous lipid components	no ω-alicyclic fatty acids (*personal communication Brian J. Tindall*)	Large amounts (>80%) of ω-alicyclic fatty acids with six- or seven carbon rings, such as ω-cyclohexane undecanoic acid -C_17:0_ and ω-cyclohexane tridecanoic acid -C_19:0_. Three species do not possess these fatty acids. Some strains are known to form hopanoids.	cellular fatty acids: ai-C_15:0_, i-C_15:0_, ai-C_15:0_; no ω-alicyclic fatty acids
oxidase/catalase	weak/weak	±/±	±/±
Inclusion bodies	poly-β-hydroxybutyric acid when grown autotrophically under ammonium starvation	not specified	some species
energy metabolism	grows best under autotrophic conditions, chemolithoautotrophic with H_2_ and CO_2_, but also chemoorganoheterotrophic; does not metabolize sugars	chemoorganotrophic or mixotrophic; carbohydrates, organic acids and amino acids can be utilized. Mixotrophic species utilize Fe^2+^ and S^0^	chemoorganotrophic

On the basis of the above-mentioned physiological, chemotaxonomic and phylogenetic characteristics of strain T2^T^, a novel genus is proposed, *Kyrpidia*, as the second genus in the parent family *Alicyclobacillaceae*, and a novel species is proposed, *Kyrpidia tusciae* sp. nov., comb. nov. Comparative characteristics of strain T2^T^ are given in Table 1.

Furthermore the phylogenetic analysis as shown in Figure 1 clearly supports the assignment of the genus *Tumebacillus* to the family *Alicyclobacillaceae*.

### Emended description of the family *Alicyclobacillaceae* da Costa and Rainey 2010.

The description of the family *Alicyclobacillaceae* is given by da Costa and Rainey 2010 in [54]. Acid may be produced from carbohydrates or not. The family is comprised of the genera *Alicyclobacillus*, *Tumebacillus* and *Kyrpidia*.

### Description of *Kyrpidia* gen. nov.

*Kyrpidia* (Kyr.pi*´*di.a N.L. fem. n. *Kyrpidia* named in honor of Nikolaos C. Kyrpides, a Greek-American genomics scientist, who co-initiated the Genomic Encyclopedia of *Archaea* and *Bacteria*).

Cells are straight rods, 1.5 to 5 μm long, facultatively anaerobic, Gram-positive, chemolitooautotrophic or chemoorganoheterotrophic. Thermoacidophilic; growth occurs above 42°C and below 67°C, with an optimum at 55°C, and at pH 4.2-7.5. Endospores are formed, but *sspE* genes for acid-soluble spore proteins are not found. The predominant menaquinone is MK-7. Major fatty acids are *iso*-C_15:0_ and *iso*-C_17:0;_ ω-alicyclic fatty acids are not present. The mol% G + C content of the type strain of the type species is 59.11 mol%. The type species is *Kyrpidia tusciae*. *Kyrpidia* is a member of the *Alicyclobacillaceae*.

### Description of *Kyrpidia tusciae* (Bonjour & Aragno 1984) comb. nov.

*Kyrpidia tusciae* (*tus´ci.ae* L. gen.n. *tusciae* from Tuscia; named after Tuscia, a region in central Italy where the Etruscians (Tuscii) lived and where the organism was found.)

Basonym: *Bacillus tusciae* Bonjour and Aragno 1984.

The genus *Kyrpidia* is comprised of one species *Kyrpidia tusciae.* The characteristics of the species are given in the genus description and the description given by Bonjour and Aragno [1].

The type strain is T2^T^ (= DSM 2912 = NBRC 15312).
